# Supplementary material for: Sediment transport drives tidewater glacier periodicity
Source: Nat Commun. 2017 Jul 21;8:90. doi: 10.1038/s41467-017-00095-5 (PMC5522421; doi:10.1038/s41467-017-00095-5)
Supplement: Supplementary file 1 — Supplementary Information [file 41467_2017_95_MOESM1_ESM.pdf]

File name: Supplementary Information
Description: Supplementary Table, Supplementary Figures and Supplementary Notes

File name: Supplementary Movie 1
Description: The evolution of glacier geometry for the temperate climate experiment over 3000 years of simulation time. The animation shows the strong hysteresis between the slow advance phase and the fast retreat phase.

File name: Supplementary Software
Description: The python script used to run all experiments (configured for the baseline temperate experiment) is included in Supplementary Software.py. The authors encourage readers to study and utilize this code base for verification or further experimentation

File name: Peer Review File
Description:

Supplementary Table 1: Table of parameters and constants for initial temperate experiment. They are valid for other experiments unless specified.

Symbol	Value	Unit	Description
ρ	917	kg m^{-3}	Ice density
ρ_r	2750	kg m^{-3}	Bedrock density
ρ_s	1600	kg m^{-3}	Sediment density
ρ_w	1029	kg m^{-3}	Saltwater density
L_f	3.35	kJ kg^{-1}	Latent heat of fusion
g	9.81	m s^{-2}	Gravitational accleration
n	3		Glen's flow law exponent
A	10^{-16}	Pa^{-n} yr^{-1}	Viscosity prefactor for ice flow
β^2	6×10^3	Pa$^{\frac{n-1}{n}}$ m$^{\frac{-1}{n}}$ yr$^{\frac{1}{n}}$	Basal traction
L	45	km	Characteristic length scale
χ	5	km	Width scaling
A_s	100	m	Bedrock undulation height
B_{max}	2200	m	Maximum elevation
B_{min}	-300	m	Minimum elevation
f	2		Mass balance shape factor
κ	1		Width shape factor
p	0.3		Topographic shape factor
W_{max}	14	km	Maximum width
W_{min}	3	km	Minimum width
b	10^{-8}	Pa^{-1}	Bedrock erosivity
c	2×10^{-12}	yr	Fluvial erosion time scale
h_{eff}	0.1	m	Characteristic water depth
$\hat{w}$	500	m yr^{-1}	Settling velocity
θ	0.5		θ-method parameter
Δx	75	m	Element size
Δt	30	d	Time step

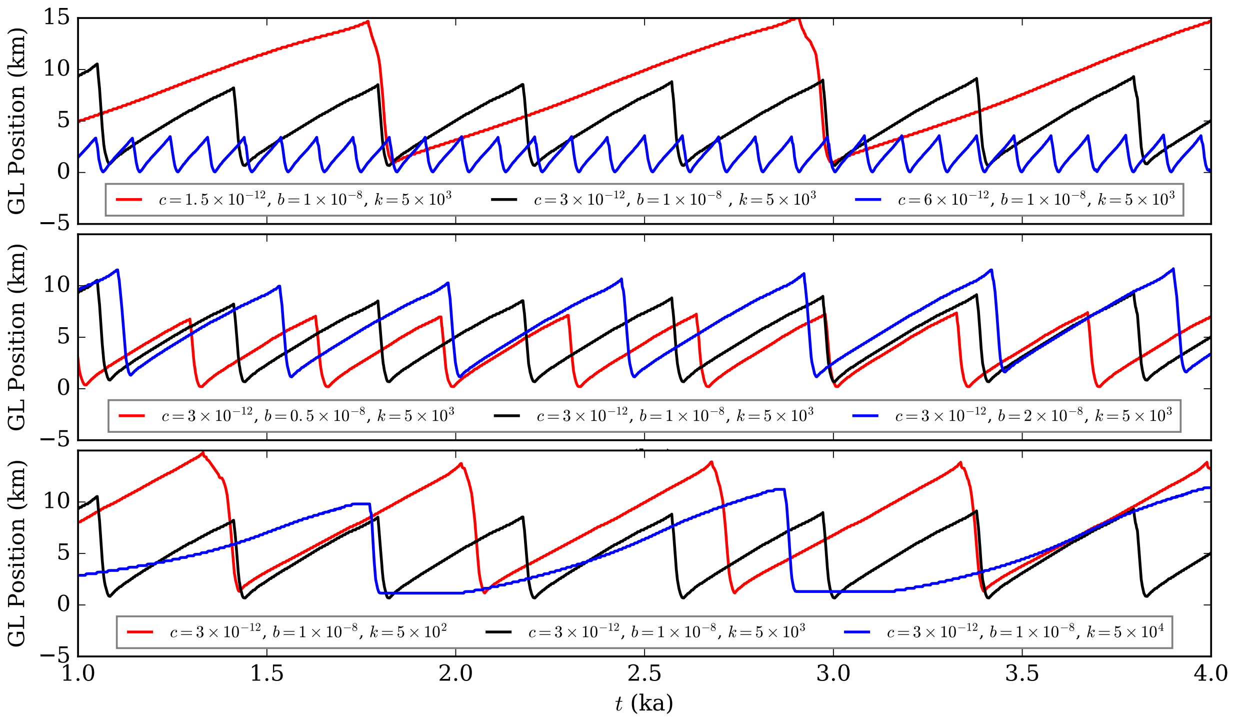

Supplementary Figure 1: Grounding line position simulated through time with different choices of fluvial erosivity (a) and bedrock erosivity (b).

Supplementary Note 1: Model sensitivity to select parameters

We ran the temperate experiment with both doubled and halved values of fluvial erosivity c and bedrock erosivity b to assess the model's sensitivity to our choices of erosion parameters (Fig. S1). We held the bedrock elevation fixed to eliminate the influence of potential nonlinear interactions between sediment cover and erosion. While the qualitative behavior of the model remains the same when varying both parameters, namely the existence of periodic behavior, the period and amplitude of the tidewater glacier cycle is quite sensitive.

Fluvial erosivity controls both the amplitude and period of cycles. Doubling the fluvial erosivity yields to an approximate halving of cycle amplitude, and period is reduced to around 30%. The physical mechanism which drives this dependence is the rate at which the terminal shoal can outpace the advance of the glacier. When sediment is easily erodible, the glacier advances faster, and the tidewater glacier instability is triggered earlier, leading to both more frequent and lower amplitude cycles. This dependence corresponds with our results showing a similar dependence on climate: more meltwater leads to more erosion and a similar decrease in period and amplitude.

An increase in bedrock erosivity corresponds to an increase in sediment supply. The model is much less sensitive to this parameter, as large portions of the bed are often covered with sufficient sediment to suppress bedrock erosion, regardless of erodibility. However, enhanced erosion near the terminus but upstream from where the shoal shelters the bedrock from erosion produces extra sediment that can bolster the shoal. This delays onset of flotation and allows the ice to remain coupled to the shoal longer, allowing a modest increase in both amplitude and period. It is interesting to note that unlike variations in fluvial erosivity, variations in bedrock erosivity do not change the rate of grounding line advance, only the extent.

We also evaluated the model's sensitivity to changes in sediment diffusivity k, trying endmember cases with diffusivity changed by one order of magnitude in either direction (Fig. S1). Interestingly, the moderate

value of diffusivity (which we used for all plots in this paper) generated TGCs with the minimum period. To understand this, we can observe the ways in which diffusion might affect the transport of the shoal and the stability of the glacier. First, in the absence of diffusion, no sediment is transported away from the moraine by hillslope processes. Because of this, sediment remains quite close to the glacier terminus, forming a steep and thick shoal that the glacier can easily recycle leading to a faster advance. In this case, the shoal also breaches the water surface quickly. Conversely, when diffusivity is high, sediment is transported away from the front, slowing advance. However, the diffusion of sediment upstream also provides a stabilizing mechanism when it can overcome fluvial transport, filling in voids and delaying flotation. In the intermediate case, diffusion is sufficient to transport sediment away from the moraine on the downslope side, but insufficient to overcome fluvial erosion and fill in nascent voids. In these high diffusion simulations, the shoal did not breach the water surface.

In the highly diffusive case, the glacier remains is in the retracted stage for long periods. This is because the moraine needs to become sufficiently wide such that sediment deposited at the terminus is not immediately diffused away by large local slopes. If diffusion is increased by yet another order of magnitude, characteristic periodicity increase to > 20 kyr, with advance ocurring only when closed basins become filled. At these time scales, we expect the myriad processes that we do not model here (e.g. uplift, isostasy) to become dominant.

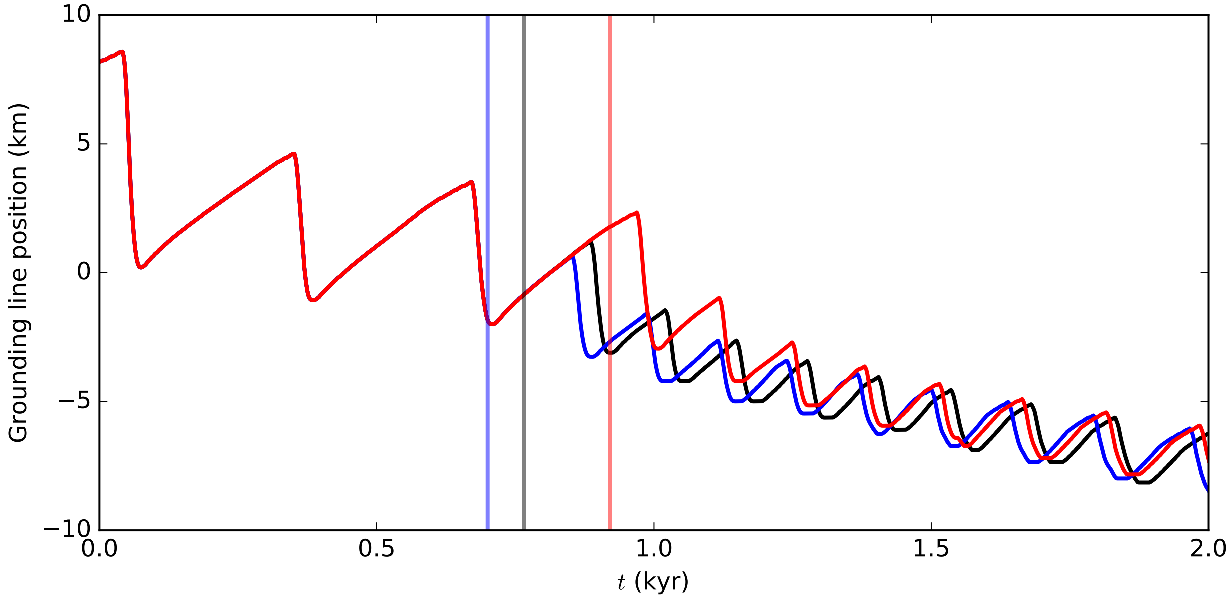

Supplementary Figure 2: Grounding line position simulated with different choices of climate perturbation timing. Perturbation is applied coincident with the vertical line of the corresponding color.

Supplementary Note 2: Model sensitivity to perturbation timing

We applied a negative climate perturbation to the temperate experiment at both the beginning of a cycle and at the end of one in order to determine whether the conclusions presented in the main text are sensitive to the choice of perturbation timing (Figure 2). When the climate perturbation is applied at the beginning of the TGC, advance still occurs, albeit with a period and amplitude consistent with the new climate. Similarly, when perturbed at the end of the cycle, the glacier finishes its 'normal' TGC before restarting with changed amplitude and period. These results imply that the conclusion that a glacier may still advance in a warming climate is insensitive to the timing of the perturbation.

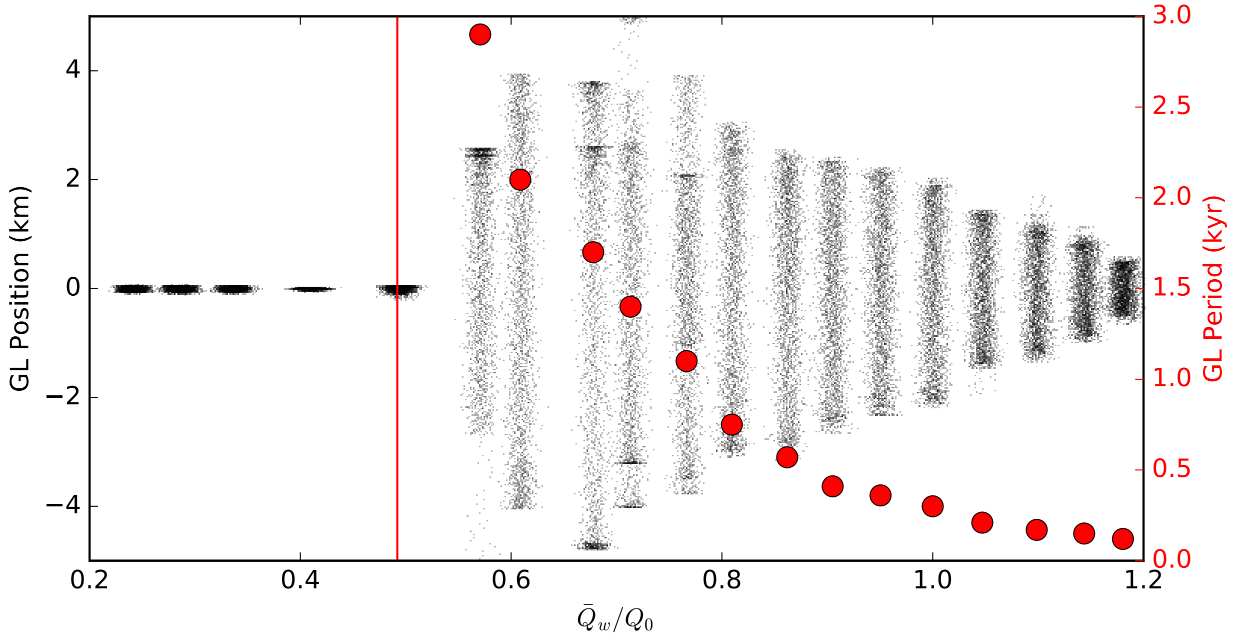

Supplementary Figure 3: Left axis: Detrended grounding line position and grounding line period at each time step as a function of time and space averaged meltwater availability $\bar{Q}_\mathrm{w}$ relative to the temperate experiment Q_0. The abscissa is perturbed by a small random factor to allow visualization of point densities.

Supplementary Note 3: Functional relationship between TGC and meltwater availability

Our results show that the availability of meltwater for fluvial sediment transport determines whether the tidewater glacier cycle will manifest in a given system. To quantify this dependence more fully, we ran a suite of experiments in which we apply a constant multiplier to the climate defined in the temperate experiment, reflecting the fact that polar environments typically have both less melt and less accumulation than temperate climates. All other things being equal, in a terrestrial environment, this should lead to glaciers with similar terminus locations. In a tidewater environment, this should lead to similar grounding line positions, although with different fluxes across the grounding line. However, in each case meltwater availability is very different. Figure 3 demonstrates a distinct qualitative shift as the advective transport due to meltwater overcomes a threshold set by diffusion (See Supplementary Discussion 1), leading to the onset of advance and periodicity. The amplitude of the TGC, as quantified by variations in grounding line position depends upon the meltwater availability: more meltwater generally leads to a lower amplitude TGC. Higher melt rates also yield a TGC with a shorter period, and this dependence is stronger than that of the amplitude. For example, a glacier with 75% of the meltwater available relative to the temperate experiment yields a TGC with roughly double the spatial extent, and with nearly quadruple the period. Additionally, while the amplitude dependence on melt seems to flatten (or even become positive) near the bifurcation point, the period increases exponentially as the threshold approaches.
